# Identification of LincRNA from *Dermatophagoides farinae* (Acari: Pyroglyphidae) for Potential Allergen-Related Targets

**DOI:** 10.1590/1678-4685-GMB-2019-0243

**Published:** 2020-03-09

**Authors:** Ying Zhou, Meili Wu, Hanting Zhu, Junjie Shao, Chang Liu, Yubao Cui

**Affiliations:** ^1^ Department of Pediatrics Laboratory, Wuxi Children’s Hospital, Wuxi, China.; ^2^ Department of Clinical Laboratory, Wuxi People’s Hospital Affiliated to Nanjing Medical University, Wuxi, China.; ^3^ Chinese Academy of Medical Science, Institute of Medicinal Plant Development, Beijing, China.

**Keywords:** House dust mites (HDMs), Dermatophagoides farinae, long noncoding RNAs (lncRNAs), allergen, RNA-seq

## Abstract

Long noncoding RNAs (lncRNAs), especially their important subclass of long intergenic noncoding RNAs (lincRNAs), have been identified in some insects. They play important roles in the regulation of biological processes, such as immune response or cell differentiation and as possible evolutionary precursors for protein coding genes. House dust mites (HDMs) are recognized as allergenic mites because allergens are found in their feces and bodies. *Dermatophagoides farinae* is one of the most important pyroglyphid mites because of its abundance in the household. To determine if lincRNAs can regulate allergen presentation in HDMs, we analyzed RNA-seq data for HDMs. We identified 11 lincRNAs that are related to mRNAs coding for allergens in HDMs. Using qRT-PCR, we amplified 10 lincRNAs and their putative target allergen-encoding mRNAs, confirming expression of these lincRNAs and allergen genes. The results suggest that lincRNAs might be involved in the regulation of allergen production in HDMs and might represent potential acaricidal candidates to inhibit mite allergen production.

## Introduction

House dust mites (HDMs) are one of the most important worldwide causes of allergic diseases, including allergic asthma, allergic rhinitis, and atopic dermatitis. Common species of HDM, like *Dermatophagoides pteronyssinus*, *Dermatophagoides farinae* (*D. farinae*), and *Blomia tropicalis* (Calderon *et al.*, 2015), produce allergenic proteins, i.e., that bind IgE from sera of >5% of patients with symptoms of HDM allergies (Caraballo 2017; Thomas, 2018). To date, 39 groups of mite allergens have been reported. The World Health Organization and International Union of Immunological Societies (WHO/IUIS) subcommittee on allergen nomenclature has assigned official names for isolated allergens, which are listed at www.allergen.org. HDM allergens are present in mite bodies, secreta, and excreta, with feces being the major source of allergens.

Production of allergenic proteins is likely regulated by both transcriptional and post-translational processes. One potential source of regulation is long noncoding RNA (lncRNA), a class of abundant noncoding RNA that overlaps traditional coding genes and includes not only antisense, intronic, and intergenic transcripts but also pseudogenes and retrotransposons. lncRNAs are involved in various processes, such as *cis* and/or *trans* regulation of transcription, dosage compensation, imprinting, and competition with other endogenous RNAs (Li *et al.*, 2014; Wang *et al.*, 2018).

A limited number of insect genes has been experimentally annotated as lncRNAs. A 2012 study identified long non-coding intergenic RNAs (lincRNAs, a subset of lncRNAs transcribed from intergenic DNA) in insects using RNA-seq data, reporting 1,119 candidate lincRNA loci in the *Drosophila melanogaster* genome (Young *et al.*, 2012). Some lncRNAs in *Plutella xylostella*, a pest of cruciferous plants, have been investigated in the context of insecticide resistance and might be involved in detoxification processes (Etebari *et al.*, 2015). In addition, a relatively high-depth screen of 35 publicly available RNA-seq datasets from *Aedes aegypti* discovered 3,482 putative lincRNAs (Etebari *et al.*, 2016). Finally, comparison of 14,161 lncRNAs from seven different species, including 1,559 from *Tribolium castaneum*, 2,602 from *Drosophila melanogaster*, 2,066 from *Anopheles gambiae*, 1,529 from *Apis mellifera*, 2,459 from *Apis cerana*, 2,176 from *Nasonia vitripennis*, and 1,770 from *Drosophila pseudoobscura*, showed low sequence conservation among different species. Further, similarities within a species are due to lncRNA association with transposable elements (TEs) and simple repeats, and TEs are less frequent in lincRNA exons than in introns, indicating that TEs may have been removed by selection (Lopez-Ezquerra *et al.*, 2017).

To our knowledge, no lncRNAs have been explored in HDMs. Determining if HDMs contain lncRNAs will contribute to the understanding of allergen expression and regulation in HDMs. Previously, we used RNA-seq to identify HDM genes (Lin *et al.*, 2018). Here, we identified *D. farinae* lncRNAs from RNA-seq data and confirmed their expression by qRT-PCR. Results of the current research will facilitate future studies to unravel the function of lncRNAs in HDMs and may help develop new methods to control HDMs or new immunotherapeutic strategies to treat allergies to mites.

## Materials and Methods

### Mite culture and transcriptomic analysis

*D. farinae* mites were isolated and cultured, and a cDNA library was constructed and sequenced according to previously reported methods (Lin *et al.*, 2018). Briefly, total RNA extracted from ~2,000 homogenized mites was used to construct a transcriptome library with a SMART cDNA Library Construction Kit (American Clontech Corporation, #634901). Sequencing was performed using an Illumina HiSeq 2500 sequencer, followed by de novo assembly with the Trinity/Oases suite.

### Total lincRNA and allergen-related lincRNA identification

LincRNAs and their targeted mRNAs were identified from RNA-seq data using a computational pipeline (Li *et al.*, 2014). To identify lincRNAs and mRNAs related to potential allergens, the sequences of all genes encoding known allergens were downloaded from www.allergen.org, the official site for systematic allergen nomenclature. Sequences were then searched against mRNAs and lincRNAs using BLASTN with a cutoff e-value of 1e-5. Allergen sequences were selected and clustered.

### Validation of lincRNA from *D. farinae*

Strand-specific real-time RT-PCR was used to quantify lincRNAs and target mRNAs coding for mite allergens. In brief, the miRNeasy Micro Kit (Qiagen, #217084) was used to isolate total RNA (TaKaRa Biotech, Dalian, China, #D312), and the PrimeScript^TM^ RT reagent kit (TaKaRa, #RR037A) was used for reverse transcription. The reverse transcription reaction system included 3 μL of total RNA (1,000 ng), 2 μL of 5X PrimeScript Buffer, 0.5 μL of RT primer (final concentration 100 nM), 0.5 μL of PrimeScript RT Enzyme Mix I, and DEPC-treated ddH_2_O to a final volume of 10 μL. Reverse transcription was carried out in an ABI 9700 PCR thermocycler at 42 ºC for 15 min, 85 ºC for 5 s, and then held at 4 ºC. A SYBR Green PCR kit (TaKaRa, #RR820A) was used for real-time PCR, performed in a total volume of 20 μL containing 10 μL of 2X Real-time PCR Master Mix, 2 μL F primer (final concentration 1 μM), 2 μL R Primer (final concentration 1 μM), 2 μL cDNA template, and 4 μL ddH_2_O. Reactions were carried out on the LightCycler 480 real-time PCR instrument (Roche) at 95 °C for 30 s, followed by 40 cycles at 95 °C for 10 s and 60 °C for 50 s. cDNA was denatured at 95 ºC, followed by 40 cycles of 95 ºC for 10 s and 60 ºC for 50 s. Melting curve analysis was performed to validate generation of expected PCR products. The setting was 65 ºC to 95 ºC at a rate of 0.2 °C per 10 s. Product was detected by incubation at 95 ºC for 5 s, 65 ºC for 15 s, and then held at 95 ºC. Primer sequences for real-time RT-PCR are shown in Table 1. Data for real-time RT-PCR were expressed using the 2^-DDCt^ method and analyzed in Excel.

**Table 1:** Oligonucleotide sets for strand-specific real-time RT-PCR using tagged primers to quantify lncRNA and targeted mRNA encoding mite allergens

Gene symbol or GenBank accession number	Primer name	Primer sequence^*^
TRINITY_DN46653	11-RT	GGCCGTCATGGTGGCGAATCATCGGAAACTATATCGAATGATTC
	11-R	GGCCGTCATGGTGGCGAAT
	11-F	ATCAGTGTTTTACCTTCCGTAT
KM010005.1	12-RT	GCTAGCTTCAGCTAGGCATCCCATTACGTGTATTACATTTAACC
	12-R	GCTAGCTTCAGCTAGGCATC
	12-F	CGTCAATTACGGCATCATTA
TRINITY_DN13286	21-F	TCTGCCCTCGATATTCTGA
	21-R	TTGATTCCTTGGATATTGCAC
KM009996.1	22-F	ATGGCTGATTTAAGACCAC
	22-R	CTTTATCTTTCTTTACTTGGCTA
TRINITY_DN20632	31-RT	GGCCGTCATGGTGGCGAATTCATCAAGCGTAACACTACTGTCCC
	31-F1	GAAGGTAACTTCGATTTGTGG
	31-R	GGCCGTCATGGTGGCGAAT
KC305502.1	32-RT	GCTAGCTTCAGCTAGGCATCGTTGGTCTTGCCAGTGCCCTTCTC
	32-F	TGAGCGTGCTCGCACCAA
	32-R	GCTAGCTTCAGCTAGGCATC
TRINITY_DN39419	41-F	TTATTGGCTGCAAACACTTG
	41-R	TCTGATCCTTGTGGTGGC
D63858.1	42-F	TTATTGGCTGCAAACATTTT
	42-R	TCTGATCCTTGTGCTGGT
TRINITY_DN51892	51-F	GCTCTATATATCATCGGTTGC
	51-R	ATAAATAATAAAATGAAATG
AY283280.1	52-F	TCACAATCTAAAATGGCACT
	52-R	ATAAAATTGGGTATGATACGAT
TRINITY_DN55962	61-RT	GGCCGTCATGGTGGCGAATGATCAACATTGACAAAGTGTTCG
	61-F	AGATCAACTCAAGACGAAA
	61-R	GGCCGTCATGGTGGCGAAT
KM009994.1	62-RT	GCTAGCTTCAGCTGAGCCGATCCACATTACACAAAGTGTTA
	62-R	GCTAGCTTCAGCTGAGCC
	62-F	GGATGCATGTAAAGGTCGT
TRINITY_DN43505	71-F	CTATATGAATAAGCGATCCAAC
	71-R	TTTCCGACAATTAATTCGTTC
KC669700.1	72-F	TATCGTTTAGTTCGTGCAT
	72-R	TATATTCAATAGTTCGCTCGT
TRINITY_DN55882	81-RT	GGCCGTCATGGTGGCGAATCAACGGGTTAATAAATTTGAT
	81-F	CGGCCACTTTTATCCTCTT
	81-R	GGCCGTCATGGTGGCGAAT
AF465625.1	82-RT	GCTAGCTTCAGCTAGGCATCTATCCTCTTCCAAATCATCT
	82-F	GGAAGGTGATGAAAGTGTTG
	82-R	GCTAGCTTCAGCTAGGCATC
TRINITY_DN54769	91-RT	GGCCGTCATGGTGGCGAATTGTCGATGGACATCTTATCA
	91-F	CATCGTTCGGTCTTTCGTT
	91-R	GGCCGTCATGGTGGCGAAT
AF178772.1	92-RT	GCTAGCTTCAGCTAGGCATCTTATTCGCCTATACAAGTCA
	92-F	AACACCAGCCCCTACAACAT
	92-R	GCTAGCTTCAGCTAGGCATC
TRINITY_DN8214	101-F	CACGTATGCAAAGATAGCAG
	101-R	AAAGAGGCAAAAGATACAGA
KM010014.1	102-F	TATTGAAGTTGAAACTACTGGC
	102-R	TTTATAAACACCGACAAGAGC
TRINITY_DN55999	111-F	AAAAAACTGTCAATCAAT
	111-R	TCATCATCATTGTAGG
KC305503.1	112-F	AAAAAACAGTCAATCAGG
	112-R	TTACGATGAATGCAAT

* RT primers contained six random bases.

## Results

### Computational identification of *D. farinae* lincRNA

We used a bioinformatics pipeline to identify lincRNAs from a set of strand-nonspecific RNA-seq data generated for *D. farinae*. From a total of 186,273 transcripts, 126,664 lincRNAs were predicted. We searched all known allergenic *D. farinae* proteins to find related lincRNAs. Identified lincRNAs and mRNAs were used to search against known allergenic *D. farinae* proteins. A total of 11 lincRNAs and 11 mRNAs related to mite allergens were expressed, some with levels indicating possible coordination (Table 2). These allergens were heat shock protein 70, ferritin, Der f 3, Der f 15, Der f 16, Der f 20, Der f 24, Der f 26, Der f 30, Der f 31, and Der f 33. Sequences for the 11 related lincRNAs are shown in Data S1.

**Table 2:** lncRNA and targeted mRNA encoding mite allergens.

mRNA GenBank Accession Number	mRNA	lncRNA	Identity	Length	Mismatch	Gap	Allergen start	Allergen end	lncNAT start	lncNAT end	E-value	Score
KM010005.1	Der f 33	TRINITY_DN46653	99.62	1057	4	0	1	1057	1060	4	0	2022
KM009996.1	Der f 30	TRINITY_DN13286	94.55	55	3	0	429	483	1	55	4.00E-16	85.7
KC305502.1	Heat shock protein 70	TRINITY_DN20632	81.69	344	60	3	1207	1547	352	9	1.00E-37	159
D63858.1	Der f 3	TRINITY_DN39419	98.12	638	12	0	1	638	29	666	0	1128
AY283280.1	Der f 26	TRINITY_DN51892	100	48	0	0	147	194	9	56	7.00E-19	95.6
KM009994.1	Der f 20	TRINITY_DN55962	82.38	227	36	4	244	468	404	628	1.00E-20	101
KC669700.1	Ubiquinol-cytochrome c reductase binding protein-like protein	TRINITY_DN43505	99.28	138	1	0	827	964	1	138	4.00E-70	266
AF465625.1	Der f 16	TRINITY_DN55882	100	265	0	0	1371	1635	287	23	4.00E-148	525
AF178772.1	Der f 15	TRINITY_DN54769	98.61	216	3	0	1458	1673	537	322	1.00E-111	404
KM010014.1	Der f 31	TRINITY_DN8214	100	54	0	0	1	54	411	464	9.00E-23	107
KC305503.1	Ferritin	TRINITY_DN55999	87.05	139	18	0	308	446	1	139	2.00E-30	133

### Strand-specific real-time RT-PCR identification of lincRNAs and mRNAs related to potential *D. farinae* allergens

**Figure 1:**
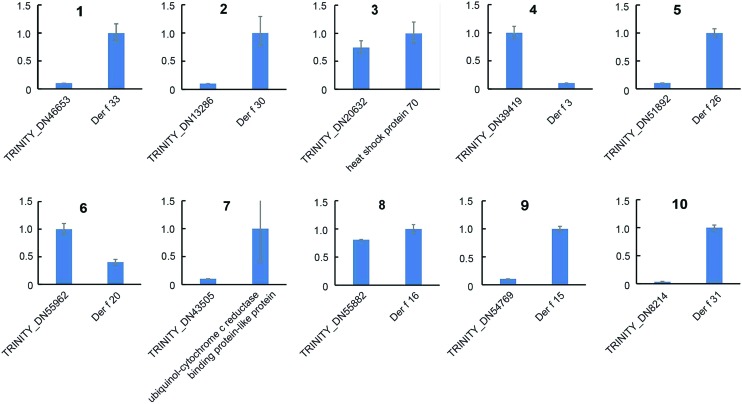
qRT-PCR validation of expression of lincRNAs and mRNAs coding for allergens: (**1**) TRINITY_DN46653 and Der f 33; (**2**) TRINITY_DN13286 and Der f 30; (**3**) TRINITY_DN20632 and heat shock protein 70; (**4**) TRINITY_DN39419 and Der f 3; (**5**) TRINITY_DN51892 and Der f 26; (**6**) TRINITY_DN55962 and Der f 20; (**7**) TRINITY_DN43505 and Der f 24; (**8**) TRINITY_DN55882 and Der f 16; (**9**) TRINITY_DN54769 and Der f 15; and (**10**) TRINITY_DN8214 and Der f 31.

Strand-specific real-time RT-PCR was used to amplify the 11 lincRNAs and their associated mRNAs from *D. farinae* total RNA. Although one lincRNA (TRINITY_DN55999) failed to generate a curve, the other 10 lincRNAs and their putative target allergen-encoding mRNAs successfully amplified (Figure 1). Low levels of Der f 3 and Der f 20 mRNA were measured in the presence of higher levels of their corresponding lincRNAs, TRINITY_DN39419 and TRINITY_DN55962, indicating a possible negative interaction. However, mRNA expression levels of Der f 15, Der f 16, Der f 24, Der f 26, Der f 30, Der f 31, Der f 33, and heat shock protein 70 positively associated with expression of their corresponding lincRNAs.

## Discussion

The present study is the first to identify lincRNAs from HDMs and to explore their potential functions. We used our previously reported RNA-seq data from *D. farinae* (Peng *et al.*, 2018) to generate a computational pipeline (Li *et al.*, 2014) that identified 126,664 lincRNAs from 186,273 predicted transcripts. To determine how many of these lincRNAs are related to HDM allergens, we searched mRNA sequences coding for 37 groups of mite allergens using lincRNAs as query sequences. A total of 11 lincRNAs corresponding to 11 mRNAs related to mite allergens were identified. Although the gene encoding ferritin and its lincRNA could not be amplified, strand-specific real-time RT-PCR identified the other 10 lincRNAs and their allergen-related potential target mRNAs in total mite RNA.

Real-time RT-PCR results showed that mRNA expression levels of Der f 3 and Der f 20 negatively correlated with expression of their corresponding lincRNAs, but mRNA expression levels of Der f 15, Der f 16, Der f 24, Der f 26, Der f 30, Der f 31, Der f 33, and heat shock protein 70 positively associated with expression of their lincRNAs. According to a previous report, recombinant Der f 33 reacts to the serum of patients with mite allergies, with a 23.5% positive rate for the skin prick test. In an asthma mouse model, Der f 33 induces airway allergy-like responses (Wang *et al.*, 2016). Further, immunoblotting showed that 63.4% of dust mite allergic patients react to Der f 26 (An *et al.*, 2013). Enzyme immunoassays indicated that recombinant Der f 16, prepared using an *Escherichia coli* expression system, binds IgE from 47% (8/17) of mite-allergic patients (Kawamoto *et al.*, 2002). In addition, 43 HDM-allergic patients have shown 32.5% positive responses to skin prick tests for recombinant Der f 31 (Lin *et al.*, 2018).

Aberrant expression of lncRNAs has been reported in immune-related diseases, including allergic diseases. For example, patients with eosinophilic esophagitis, an allergic inflammatory disorder, have altered lncRNA profiles (Sherrill *et al.*, 2014), and altered lincRNA expression has been identified in CD8+ T cells of severe asthma patients (Tsitsiou *et al.*, 2012). lincRNAs are involved in the innate immune response, including activation of monocytes and macrophages as well as regulating expression of inflammatory genes (Hadjicharalambous and Lindsay, 2019), highlighting potential pathways through which lincRNAs may mediate allergenic responses. However, few studies have examined lncRNAs with regards to HDM allergens. Our previous results show that exposing cells to HDM extracts results in differential expression of 270 lncRNAs, 119 of which were co-expressed with mRNAs. Bioinformatic analysis suggested these lncRNAs may target gene pathways related to glycolysis, axon guidance, ErbB signaling, and MAPK signaling (Wang *et al.*, 2018).

In this study, we identified lncRNAs related to allergens produced by the important HDM *D. farinae*. One potential application of lincRNAs is the development of acaricidal candidates to inhibit mite allergen production in homes. However, to test that possibility, we must first validate the functions of these lincRNAs by developing an asthmatic animal model sensitized by mRNA coding for allergens and then treat animals with corresponding lncRNAs. This is a logical next step in future research.

## Supplementary material

The following online material is available for this article:

Data S1 Sequences of lincRNAs identified from this study.
