# Supplementary material for: Identification of LincRNA from Dermatophagoides farinae (Acari: Pyroglyphidae) for Potential Allergen-Related Targets
Source: Genet Mol Biol. 2020 Mar 9;43(1):e20190243. doi: 10.1590/1678-4685-GMB-2019-0243 (PMC7198022; doi:10.1590/1678-4685-GMB-2019-0243)
Supplement: Supplementary file 1 [file 1415-4757-GMB-43-1-e20190243-s001.pdf]

Genetics and Molecular Biology, 2020, 43(1)

Suplementary Material to "Identification of LincRNA from *Dermatophagoides farinae* (Acari: Pyroglyphidae) for Potential Allergen-Related Targets

Data S1 - Sequences of lincRNAs identified from this study.

>TRINITY_DN46653
ACAATTGTACAAATTGAACAGTACTTTTAGCTTTTATGGCAGCAATTGCAGCATTCACATCTTTTG
GTACAACATCACCACGATATAGCAGGCAACAGGCCATATATTTACCATTACGTGTATTACATTTA
ACCATTTGATTTGATGGCTCAAAACATGTACCGGTTATTTCCGAAACAGTAAATTGTTCATGGTA
GGCTTTTTCACTCGATACAATCGGTGCATAACTAACCAATGGAAAATGTATCCTTGGATAGGGTA
CCAAATTGGTTTGAAATTCGGTCAGATCAACATTTAGTGCACCATCAAAACGTAATGATGCCGTA
ATTGACGAAACGATTTGTCCAATTAAACGATTTAAATTCATATATGATGGACGTTCAATATTTAGA
TTACGGCGACAAATATCATAAATGGCTTCATTATCAACCATAAATGAACAATCCGAATGTTCCAA
TGTATTATGCGTGGTTAATATTGAATTATATGGTTCAACAACAGCGGTTGAAATAGCAGGTGCCG
GATAAATAGCGAATTCTAATTTAGATTTTTTTCCATATTCAACCGATAAACGTTCCATTAATAATG
AAGAGAAACCTGAACCAGTACCACCACCAAATGAATGAAATATAAGAAATCCTTGTAGACCGG
AACATTGTTCAGCTAATTTTGCGATACGTCTCATAACTGGTTCGATCAGTGTTTTACCTTCCGTAT
AATGTCCACGTGCGTAATTATTAGCCGCATCTTCTTTCCCAGTGATCAATTGTTCTGGATGAAATA
GACGACGATATTCACCCGTACGTACTTCATCAACAACTGTTGGTTCTAGATCAACATAAATTGAA
CGAGGTACAAAACGCCCATTACCGGTTTCATTGAAAAATGTTGAAAATGAATCATTCGATATAGT
TTCCGATGAAGACAATGTTGTTGTCGATTTAACAGGTGATAATTGGCCATCCGGTTGAATTTCAT
GTTCCAGACAATACAATTCCCAACAGGCATTCCCGATCTGTACACCAGCTTGGCCAACATGTAA
TGATATACATTCACGCATTTTTGGTCAGTATTCTAAAAAAGTTTCAGAGTTTTAAAATTTTAAAATT
AAAACAAAACAAAAAAAATGTTTCTGGTTGACAAGCAACGAACACAACCGAAATCGAAGAACGA
ATGTTTAGTAGCTAGAAGGGCGCGTCCAATGATTTGTTCAATTTGGGTTGGTGATTGTTGACATT
TTTATCATCA

>TRINITY_DN13286
GTTCATCAAAAATGTCATGGCAGGCCCGTTCCCAGAAAAGGCAGGAAAAACATAATCAAAATA
AAAAAAAAAAAAAAAAAAAAAAAAAAAAAAAAAAAAAAAAAAAAAAAAAAAAAAAAAAAGAAAAGCCG
GATGTCTCTTTCTGCATGGACAAGTGCGATGACTGGTTCTGGACTCCGAAGTTGATTGACTGACC
TTTTCGTTATTCTGTTCTCCATCTCCTAATCATCTAGACGACGGTGGAGATGGACGGTGTTTCTGG
TGCATTTCTTGCTTCTGGCGAAGTGGTTTCCTTTCCTTTTTTTTTTTTTTTTTAATATTTCCTGTCTTT
TTCAATTTCCAGGCTGTTATTTGTTGTTATTTTATTTGCTTTGTCTACGTTGTGTGTTCTGCTCTGCC
CTCGATATTCTGATTGATCTTTTGCCGATTGTCTGCCCTTATTCAATGAAAGTGGTAGAATCAGAG
GGTTCTCTTGAAGAATGGATTTCTTGACTTATGCCACTGAGCACTGTTCTGTAAATGAGACTAGT
GTTAAAGTGCATCCAAGCCCTAGAGGATACATAGTAGCCGAGGTATCCATTTGGTTTGTCTGGT
CCCTTCTACGTACAGTGCAATATCCAAGGAATCAACCGTTCCCAC

>TRINITY_DN20632
CCATTCTTTCAATGTCTTCCTTAGAGAGACGACCCTTGTCGTTGGTGATGGTGATCTTGTTTGATT
TACCGGTAGTCTTGTCAGCAGCGTTGACGTTCAAGATACCGTTAGCGTCAATGTCGAAGGTAAC
TTCGATTTGTGGAACACCACGTGGTGCTGGTGGGATACCTGAAAGTTCGAATTTACCGAGGAGG
TTGTTGTCCTTTGTACGTGCACGTTCACCTTCGAAGACTTGGATCAAGACACCAGGTTGGTTGTC
AGCGTAAGTTGAGAAGACTTCAGACTTCTTGGTTGGGACAGTAGTGTTACGCTTGATGAGGGCA
GTCATGACACCACCAGCGGTTTCAATACCAG

>TRINITY_DN39419
GATTTTTCTTTGGCAAGCATCATAATCAATTTTTCAAATTAACAAATAAATCATATATTGAAATCG
AACCAAGATGATGATTCTTACCATTGTCGTGTTATTGGCTGCAAACACTTGGGCCACACCGATTC
TTCCATCATCACCAAATGCAACTATTGTTGGTGGTGTGAAAGCAAAAGCAGGTGATTGTCCATAT
CAAATTTCCTTGCAATCAAGCAGCCATTTTTGTGGTGGTAGTATCCTGGATGAATATTGGATCTT
GACCGCTGCACATTGTGTCAATGGACAATCAGCAAAAAAACTTTCAATTCGTTACAATACTCTTA
AACATGCATCTGGTGGTGAAAAGATTCAAGTGGCGGAAATTTATCAACACGAAAATTATGATAG
CATGACTATCGATAATGATGTTGCATTGATAAAACTCAAAACACCAATGACATTGGATCAAACA
AATGCTAAACCCGTACCATTGCCACCACAAGGATCAGATGTAAAAGTTGGTGATAAAATTCGTG
TTTCTGGTTGGGGTTATCTTCAGGAAGGAAGTTATTCATTACCATCGGAATTACAACGTGTTGAT
ATTGATGTTGTATCACGTGAACAATGTGACCAATTATATTCAAAAGCAGGCGCCGATGTTAGTGA
AAATATGATTTGCGGCGG

>TRINITY_DN51892
GTTTTTTTCGATATTGCTGCCGATAATCAACCATTGGGTCGTATTGTCATTGAGCTTATTTTTTTCT
CGGAGATTGTTTCTCGAAAACGAAGAAATTATAGCTCTATATATCATCGGTTGCTTGCTTGTCAT
GACGTGATAAGCAGCATAACCGGCGAGGTGTGCGATTTGAGATTTTCTAAATCTCAACCATTAA
ATGATGATGCCATGTCGTGTTCACGCTGTCTTGTTCTCTTTGGCTTCAGCAACGTTGTAGCCAGTT
TTTTTCTCTTTTCTGTCATATCGATTGATTGACAGGCATTGGTTGTCGATAGATGGCGCTTCTAGC
CAATGGGTGTTTTTCATTTGAAATGAAAAAAAAAATTTATTTTTAGTAAAAATTTCAACTTTTTGAT
TTTATTTTCATTTCATTTTATTATTTATTATTAGCTCCGTAGTGATGTTGTGCCCAAAACAGCG

>TRINITY_DN55962
TTACCAAATATTTGTTATTCTGTATTGTATGTGTAATTGAATTTGTTTATTTTATTTTTTTTTTGTCAC
AACTGTTGTCAACTTTTCAATGCTTTCATTTTATTCTTTGTCCATCATTGTTCGTGAATATCTTGAA
CAGTACGAATTCTGTCCGAAAAAAATGGTCGATCAAGCTACCCTGAGTAAATTGGAAGCCGGTT
TCCAAAAATTACAGAATGCTCAAGATTGTCATTCATTGTTGAAAAAGTATTTGACTCGCGATGTG
TTAGATCAACTCAAGACGAAAAAGACCGACATGGGCGCAACATTATTGGATGTTATCCAATCTG
GCGTGGAAAACCTGGACAGTGGTGTTGGTATCTATGCTCCTGATGCTCAATCATACAAAACATTT
GCTGCATTGTTCGATCCAATCATTGATGATTACCATAAAGGCTTCAAACCGACCGATAAACATCC
GCAAACTGATTTCGGCAATATCGAACACTTTGTCAATGTTGATCCTAAAAACGAATACGTCATTT
CTACTCGTGTTCGATGTGGCCGTTCGTTGAAAGGCTATCCATTCAACCCTATGTTGACAGAGGCT
CAATACAAAGAAATGGAAACCAAAGTGAAAGGACAATTGGCCACATT

>TRINITY_DN43505
ATTCCCGTTTATCTTTATCATCAATTTTCATGTTGCGATTTTTAAACATTTTAAAAAGATGATAAAC
ATTGTGCAATAATGGTATCGATATTGGTAAAAATAATGGCAAATAAATGGCATATTTTTGATCAT
CTGGAAAATAAAGTAATGATAATAATGATGGATCATAGAATGCTTGTTCACTATATGAATAAGCG
ATCCAACTTTTTTCATATGCTTCAATAATATCACCATTTGAATTCAATAATTCAGCCGCTTTCATTG
ACATTTCCATTGAATCATGTATGCGACGTGAAATTTTCTCTTCAATAACCATGTTGCTCACTTTATT
CAATAATTTTTCAATCGATTCCAATGATTTCAATGTTTCGTGGATATGTTTCTGCACCAATGCATG
AACAATGGATTCAATTTCTAATTGTGTAAAAAATTCACCCGATACTTGGCAACGATTTGGTAGAT
ATAAAAATGAACGAATTAATTGTCGGAAACCATTGAAAAGATCTTTTTCATTATTCACAATAAGT
ATTGATCCACGAAATGGTGTTGATATTAGATTTGATTGATTATTAGTTTGATTATCGAAAAATTTC
AACACTGGTAATGATTGTGATCGAACATATAGAACTAAATGATAACTTTTATCATTATCATGTTCT
ACACGGCTTGTTTCAATTCGATTCAATATGGAATCAATAGATGATATTGGTAATAATCGTTCTTGT
TGTTGTTCCGATGTATTGTTTGATGATTTCAATAATTTTGATTGAATAAAATTATCACGTGGAAAT
ACATAATGTAATACCTGTGTAATGAAATTGATACGAAAATATTGACTAAAACTAAAACGATGTGT
ATAGAAACGTTCCAATAGTTGATCAATTTGTTTGATCCATTTTCGATGACGTTCTTTACGTTCGTTT
AGATTTAAATTTTTATCCACATGATCATCTTCATAAATGATGTCAAATAAAAAATCAAAATCTCTA
GCCATCAATAGAACAATATTTTGTTCTTGTTTATCAGCTTGATGCGAATAAAAACGTGTGATGAG
CATTTGAACCTGATGAACATACTGGTTGATTTGTTCGCCCAAACATTCGACAATATTTACCTGGC
CACAGATATTATCCAAATGATTTAGATCAATAAAATAGGATCGATATCTGCCGGCATTTATAAAT
CGATCTGTATGAATATTTCTAGGTAAAATATAAAACATAATTGAATTGATTGATTGAATATTATCG
GCTGATAGCAGTTGATCCAAATCATTCAATTCAAATGAATCTTGATTTCGTATCGATTTGAATGCC
ATTAATTCGTTATTGAATGCTGATCGTACTGTCCATTCTTGACGAAATGAAAGACTATTATCAAAG
CTAATTCTTGTATGATGATTTGCATGCAGATAATTGCGGAGATCAATATTTTGGAATGATTCATCA
GATTCATTCAGATGAATTATTGATATTTTATAATAATGTGTCAATGTTTGTGATCGTACCATTAGC
CATGAAATATCTGGTAATGGCCATCGTTCGGGTGTTGTTGTACGATACCAAATCGGTAATCCAAT
CAAAAACAATACGGACAAATAGAATATGGATGCAATCAAAGTGGAACGATTCGATTGTTTTTTC
GATTTTTCTACACAATCTTTAACGATTCTTTCGAATTCTCGTTCCAATACATCATTATTTTGGTCAT
CATTGGCCATGTTGATTTAAAAACAATTTATCATCAAACAAAT

>TRINITY_DN55882
TGAAAAATTCGGATTTTTTTTTATTCAAAATTCTATAAATTCATCTCGATCAACACCAAACATGGA
TGATGATGAAATCATTTAATTCAGTTCCATTTAGGAAAATATGATTTGAATGCACCATCTTCACTT
CCTTGTTTTAGTTTTATAATCTGATTTGGTTGAAATTTACGGCCACTTTTATCCTCTTGTATAAAAC
GTTGGGCTACTTTATCAACATGTGCTTCATCGGCTATACGTTCAGCAAATTGGTTACCAATCCAA
ACATAAATCTTATCCATAACATCTTTTTTTGGAAATGAAATGAAAAAAAAAAATCAAATTGTTATAA
TTTTATCAAATTTATTAACCCGTTGATTATTATTATTATTTTGATGTTGTTATTAGAAAATTAAAATA
CACATACCCAAAATCATGATATTATCCTCT

>TRINITY_DN54769
ACCACCTTCAGCAAATCGTTGTCCCAATTTATAGCCATAATATTGTATAATTTGTGTTTCATTTGC
AAATCTTGTACTTGGTACCTGATAAAAACGTGTGAATTTATCCGCATCAATCCGATTTAGTTGTC
GTGAATCACAACATTTGCTTAAACTTCGTACTAGACCATCGTTCGGTCTTTCGTTCATGTTCACAA
ATTTTTTCGACTATTTGAATTCCCTGATTTTATACGATTTTCAAGAAAATAATTTAAATTACATAGG
AATTATTGAATTTTTCATATTAAAAATTGAATTTTAAATTTTAATTGAATTTTTTTTCAGAATTATTC
GCCTATACAAGTCAATTTTTCTTGACACCAAATAGTGCCCGGAGGACAAGGCATAATATGAACC
CACCAGCCACCATTAACAAATTCACAGACTAAATATTTGTGTATATTGGTTGGATGTGGGATATC
ACCTTCCTTGTAACATTTGATAAGATGTCCATCGACATAGGTTGTATATTTTGGTGTTTCGCTTGT
GTGTTCGGTCGTGGTTGGCGAAGGTTTCGATGTTGTAGGAGTTGGCGTTGGTGTTGTTGGTGTTG
GTGTGGTGGGAGAAGGAGTTGTTGTCGGTGTGGTGGGAGAAGGGGTTGTTGTCGGTGTGGTGG
GAGAAGGGGTTGTTGTCGGTGTGGTGGGAGAAGGGGTTGTTGTCGGGGTGGTGGGAGAAGGA
GTTGTTGGTGTTGTAGTCGGGGTTGTGGGTGTCGTCGTTGGAGTTGGTGTCGTTGT

>TRINITY_DN8214
AAGACAAAGAAAAATCAAAAATGGTAAGTGTTTTTTTTTTCTGATAAAATATAACAATTGTCAAT
GATTATAAATTTATGTTTCTGGATCGTCAATGATGATGATGATTATGATTTGAAATTCGAATTTGA
TTGACATTAACCCAACACACACAATACACACGCACGTATGCAAAGATAGCAGCCACGGTCTCTA
TCACATATAATTCAGTATAGCACTCTATTAGCTTGTTATGTGTATTATCCACCAAATGGTTTGGCC
AATATTGCCGACGCCCTCAGTACATTAGTCGTTTATAATTTTGCGGCTGTTTCTGTATCTTTTGCCT
CTTTTCATTTTTGTTTATCGTCAATTTCCACATTTTTTTCATCATTATTTGAATTGCTGAATTTTTTTT
ATATCAGAAAAAAAATGGCATCCGGAGTTACTGTCGCAACGGAAGCAAAAACGCTTTATGAAGA
AGTGA

>TRINITY_DN55999
AAAAAACTGTCAATCAATCATTGTTGGATTTACATGCTGTTGCCACTAAAAATAATGATGTTCAA
TTTGCCGATTTTCTTGAAACACATTATTTAACTGAACAAGTTGAAGCAATCAAAAAATTGGCCGA
TTATGTTACACAATTACGTCGTTGTGGTCTTGGTTTGGGTGAATATCTTTTCGATAAACATACCCT
ACAATGATGATGATAATCTTAATCATAACAAAAAAAAAAAAAAAAAAAAAAAAAAAAAAAAAACAA
AAAAAAAAAAAAAAAAAAA
